# Automated conversational agents for post-intervention follow-up: a systematic review

**DOI:** 10.1093/bjsopen/zrab070

**Published:** 2021-07-29

**Authors:** L Geoghegan, A Scarborough, J C R Wormald, C J Harrison, D Collins, M Gardiner, J Bruce, J N Rodrigues

**Affiliations:** 1 Section of Vascular Surgery, Department of Surgery and Cancer, Imperial College London, London, UK; 2 Department of Cardiothoracic Surgery, King’s College Hospital, London, UK; 3 Nuffield Department of Orthopaedics, Rheumatology and Musculoskeletal Sciences, University of Oxford, Oxford, UK; 4 Department of Plastic, Reconstructive and Burns Surgery, Chelsea and Westminster Hospital, London, UK; 5 Department of Plastic and Reconstructive Surgery, Frimley Park Hospital, Guildford, UK; 6 Warwick Clinical Trials Unit, Warwick Medical School, University of Warwick, Coventry, UK; 7 Department of Plastic and Reconstructive Surgery, Stoke Mandeville Hospital, Aylesbury, UK

## Abstract

**Background:**

Advances in natural language processing and other machine learning techniques have led to the development of automated agents (chatbots) that mimic human conversation. These systems have mainly been used in commercial settings, and within medicine, for symptom checking and psychotherapy. The aim of this systematic review was to determine the acceptability and implementation success of chatbots in the follow-up of patients who have undergone a physical healthcare intervention.

**Methods:**

A systematic review of MEDLINE, MEDLINE In-process, EMBASE, PsychINFO, CINAHL, CENTRAL and the grey literature using a PRISMA-compliant methodology up to September 2020 was conducted. Abstract screening and data extraction were performed in duplicate. Risk of bias and quality assessments were performed for each study.

**Results:**

The search identified 904 studies of which 10 met full inclusion criteria: three randomised control trials, one non-randomised clinical trial and six cohort studies. Chatbots were used for monitoring after the management of cancer, hypertension and asthma, orthopaedic intervention, ureteroscopy and intervention for varicose veins. All chatbots were deployed on mobile devices. A number of metrics were identified and ranged from a 31 per cent chatbot engagement rate to a 97 per cent response rate for system-generated questions. No study examined patient safety.

**Conclusion:**

A range of chatbot builds and uses was identified. Further investigation of acceptability, efficacy and mechanistic evaluation in outpatient care pathways may lend support to implementation in routine clinical care.

## Teaser

The COVID-19 pandemic has necessitated a shift away from in-person consultation and follow-up. The authors performed a systematic review to determine the acceptability of using chatbots to engage with patients following intervention. They identified ten studies which report the use of chatbots following intervention and outline pragmatic steps which must be taken before clinical implementation.

## Introduction

The first known agent capable of conversation between human and machine was developed in 1966^1^. *Eliza* used early natural language processing to return open-ended questions to users, simulating person-centred psychotherapy.

Developments in speech recognition, natural language processing, natural language understanding and artificial intelligence have led to the design of systems capable of mimicking human interaction with unconstrained natural language input^2^. A chatbot is defined as ‘a computer program designed to simulate conversation with human users, particularly over the internet’^3^. A recent systematic review involving 17 studies and 1573 participants found that chatbots in healthcare were predominantly used in mental health conditions to educate patients and collect data from health-related questionnaires^4^.

Financial pressures and clinical demand have driven interest in virtual clinics for monitoring and surveillance following healthcare interventions^5^, particularly during the COVID-19 pandemic, with rapid adoption of virtual services to moderate infection risk through reduction of direct clinician–patient contact^6^. A recent randomised trial involving 209 general surgical patients demonstrated better attendance (92 *versus* 81 per cent) and higher patient satisfaction (95 per cent of participants happy or very happy *versus* 56 per cent) with virtual postoperative clinics compared with traditional outpatient follow-up^7^.

Chatbots hold promise in increasing the efficiency of outpatient care pathways and meeting the need for patient surveillance and education between face-to-face clinic appointments. Accuracy of information and patient safety, however, are important considerations. The aim of this systematic review was to determine the uptake, acceptability and utility of chatbots in the follow-up of patients who have received physical healthcare interventions.

## Methods

The systematic review was designed and reported in accordance with the Preferred Reporting Items for Systematic Reviews and Meta-Analyses (PRISMA) statement^8^. The protocol was prospectively registered in the PROSPERO database (registration number: CRD42020199919)^9^.

### Search strategy

Search strategies included free text and index terms related to the following core concepts: ‘chatbot’ ‘intervention’ and ‘follow-up’ (*Fig. S1*, supplementary material). The following databases were searched from inception until 18 September 2020: MEDLINE, MEDLINE In Process, EMBASE, Cochrane CENTRAL, CINAHL and PsychINFO. The Central database was searched for registered clinical trials up until 9 November 2020. The search was not restricted by language or date of publication. A further search of the surgical grey literature was conducted by examining the proceedings of the 2020 Association of Surgeons in Training International Surgical Conference^10^^,^^11^.

### Eligibility criteria

All studies reporting original data were eligible for inclusion, including randomised trials, quasi-experimental designs, cohort studies, case-control studies and case series. Case reports, reviews, meta-analyses and articles related to the technical development of systems without accompanying clinical data were excluded. Systematic reviews were screened for potentially eligible publications. The titles and abstracts of identified articles were independently screened by two authors.

### Participants

Adult and paediatric patients who had undergone any physical healthcare intervention targeting physical rather than mental health and who were subsequently followed up using an automated conversational agent (a chatbot) at any point after an intervention were eligible for inclusion. Physical interventions were defined as procedures where purposeful access to the body was gained via an incision, percutaneous puncture or instrumentation via a natural orifice or the provision of medications to treat underlying disease. Examples of physical interventions included total hip replacement for osteoarthritis, steroid injection for carpal tunnel syndrome, transurethral resection of the prostate for benign prostatic hyperplasia and the prescription of antihypertensive medication.

### Interventions and comparators

A chatbot was defined as a computer software application that permits two-way conversation (via text, speech or a combination of both) between a human user and a computer program^3^. Comparators included other automated or non-automated follow-up systems, including, for example, routine care delivered via face-to-face outpatient clinics and follow-up telephone calls.

### Outcomes

The primary outcome assessed was the acceptability of chatbots as a method of follow-up indicated by implementation success. Measures of acceptability included user engagement (defined as the proportion of patients who activated and interacted with the chatbot), patient adherence to the chatbot, response rate (defined as the proportion of patients responding to system queries), duration of adherence and interactions with the chatbot over time. Patient safety and accuracy statistics were assessed where reported. Additional outcomes assessed included patient cohort demographics, design features such as task orientation, dialogue management, input and output formats, platforms used, health questionnaires used and measures of patient satisfaction.

### Study selection

Potentially eligible studies were compiled, and duplicate citations removed. Two authors independently screened titles and abstracts of retrieved studies using prespecified stepwise inclusion/exclusion criteria. Disagreements between reviewers were resolved through consultation with a third reviewer. Reference lists of included studies and published narrative/systematic reviews were examined for further potentially eligible studies.

### Data extraction and analysis

Data were extracted using a predefined electronic data-collection form. Extracted data were collated, cross-checked by other authors and compared. Study setting, population demographics, healthcare interventions, cohort-specific factors, software design features, measures of adherence, patient experience and clinical outcomes were extracted. Formal meta-analysis was not performed due to heterogeneous outcome reporting and differences in study designs. A narrative synthesis and descriptive analysis were used.

### Risk of bias analysis

Methodological quality of each included study was assessed. For randomised trials, this involved the revised Cochrane Risk of Bias tool^12^, and for non-randomised comparative studies the Cochrane Risk of Bias in non-randomised studies of interventions (ROBINS-I) tool^13^. The National Institute of Health (NIH) quality assessment tool for cohort studies was employed to assess the quality of cohort studies^14^.

## Results

**Fig. 1:**
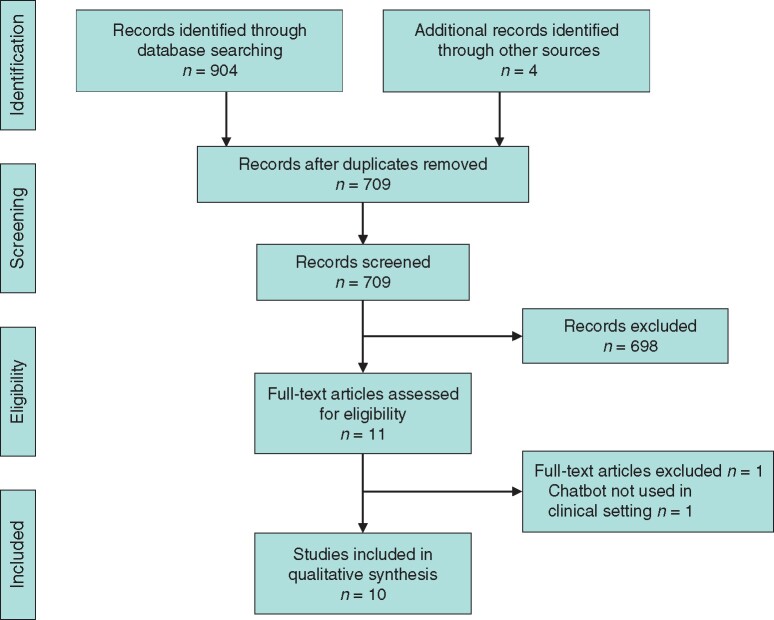
PRISMA flow diagram

From a total of 908 potential studies, 709 remained for screening after removal of duplicates, of which 11 articles were finally assessed with 10 meeting full inclusion criteria (*Fig. 1*).

### Study characteristics

Three randomised control trials (RCTs) were identified. One involving 76 participants compared an automated text-based chatbot with standard postoperative care following upper or lower extremity fracture^15^. The second, involving 142 participants, compared an automated chatbot *versus* physician-generated advice for women who had undergone breast cancer treatment^16^ and the third, with 45 participants, compared immediate *versus* delayed access to a chatbot in young patients affected by various cancers^17^.

The non-randomised comparative clinical study included 270 participants and compared an automated speech-based chatbot to manual telephone follow-up for patients who had undergone orthopaedic surgery^18^. The remaining six studies^19–24^ were cohort studies based on an established definition^25^. Collectively, eight out of 10 studies were published between 2019 to 2020.

### Demographics

Of the 10 included studies, nine recruited adults, and one adolescents with a mean age of 15 years^19^ (*Table 1*) resulting in a total of 5492 patients. Chatbots were used to follow up patients after elective orthopaedic surgery^18^, orthopaedic trauma surgery^15^, surgical intervention for varicose veins^21^, women treated for breast cancer^16^^,^^24^, uretoscopy^23^ as well as the medical management of hypertension^20^, asthma^19^ and various cancers^17^^,^^22^.

**Table 1:** Study demographics, quality and risk of bias

Reference	Study type	n	Speciality	Cohort	Intervention (+ control)	Study quality	Risk of bias
**Anthony *et al.*,** 2020^15^	RCT	76	Orthopaedics	Adult patients who had undergone operative fixation of upper or lower extremity fracture	Automated chatbot which delivered text messages to reduce opioid use *versus* standard postoperative care	–	Some concerns^*^
**Bian *et al.*,** 2020^18^	Comparative clinical study	270	Orthopaedics	Adult patients who had undergone orthopaedic intervention	Automated chatbot *versus* telephone follow-up for postoperative care	–	Moderate^†^
**Bibault *et al.*,** 2019^16^	RCT	142	Oncology	Adult patients in remission or undergoing active treatment for breast cancer	Automated chatbot which provides information to patients about breast cancer, epidemiology, treatment options, side effects and quality of life improvement strategies *versus* information provided by treating physicians in real time via text message	–	Some concerns^*^
**Black *et al.*,** 2020^21^^‡^	Prospective cohort	158	Vascular surgery	Adult patients undergoing intervention for lower extremity superficial venous reflux with endovascular ablation, sclerotherapy and phlebectomy	Automated postoperative chatbot offered to patients to educate patients, provide postoperative instructions, facilitate follow-up appointment booking and contact with the clinic	–	–
**Chaix *et al.*,** 2019^24^	Prospective cohort	4737	Oncology	Adult patients in remission or undergoing active treatment for breast cancer	Automated chatbot which provides information to patients about breast cancer, epidemiology, treatment options, side effects and quality-of-life improvement strategies	Fair	–
**Giorgino *et al.*,** 2005^20^	Prospective cohort	15	Internal medicine	Adult patients with diagnosed hypertension treated with oral medications	Automated chatbot used to monitor hypertensive patients in the community. Collects health-related data such as heart rate and blood pressure	Poor	–
**Goldenthal *et al.*,** 2019^23^	Prospective cohort	20	Urology	Adult patients who had undergone ureteroscopy for nephrolithiasis within the previous month	Automated chatbot used to educate and reassure patients regarding commonly experienced symptoms or post-procedural complications	Fair	–
**Greer *et al.*,** 2019^17^	RCT	45	Oncology	Young adult patients (aged 18–25 years) who had completed active treatment for cancer within the past 5 years	Automated chatbot used to provide cognitive and behavioural intervention that develops eight positive psychological skills. Patients were given conversational teaching sessions and practice lessons. Control participants were asked to provide daily emotion ratings. The control group had no access to the chatbot but were given access after 4 weeks	–	High^*^
**Piau *et al.*,** 2019^22^	Prospective cohort	9	Internal medicine	Adult patients aged >65 years with a diagnosis of cancer undergoing active treatment with chemotherapy	Automated chatbot used to identify the development of symptoms or treatment side effects	Fair	–
**Rhee *et al.*,** 2014^19^	Prospective cohort	15	Internal medicine	Adolescent patients (and patient dyads) diagnosed with asthma receiving active treatment	Automated chatbot used to monitor patient symptoms, activity levels and medication use	Fair	–

* As per RoB 2 tool.

† As per ROBINS-I tool.

‡ Quality appraisal and risk of bias assessment not performed as full manuscript not published (data extracted from conference proceedings).

### Quality of included studies

One RCT was deemed to have a high risk of bias due to ascertainment bias and risk of detection bias given the effect of unblinding on the outcome of interest^17^. The remaining two RCTs were deemed at moderate risk of conduct bias^15^^,^^16^.

The cohort studies were rated as fair^19^^,^^22^^,^^23^ or poor quality^20^ (*Fig. S2a–c*, supplementary material). The quality of outcome measurement and assessment was deemed poor across all cohort studies.

### Interventions

All studies deployed chatbots on mobile devices: two were also accessible via web-based applications^16^^,^^24^ and one was accessible via Facebook Messenger^17^. In terms of chatbot construct, seven used a frame-based knowledge-representation system^16–20^^,^^23^^,^^24^, one used a rule-based knowledge-representation system^22^ and two studies did not report the type of system used^15^^,^^21^. Of the 10 studies, three used a system-focused dialogue^15^^,^^22^^,^^23^, two a user-focused dialogue^16^^,^^24^ and the other five used a mixed dialogue initiative^17–21^. Task orientation was reported in two studies, one chatbot was able to book follow-up appointments^21^ and one was able to input patient data into electronic medical records^20^.

### Outcomes

Measures of implementation success were reported in seven of 10 studies^17–19^^,^^21–24^. Adherence ranged from 31 per cent participant engagement rate^24^ to 97 per cent participant response rate for select system-generated questions^19^. One study demonstrated a decline in engagement from 100 to 31 per cent after 8 months of chatbot use^24^. A comparative study demonstrated a 92 per cent follow-up rate for patients contacted via an autonomous postoperative chatbot versus a 93 per cent follow-up rate for patients contacted directly by phone^18^.

Other outcome measures reported by studies included patient-reported outcome scores (PROMs), patient feedback, patient experience and technical details related to chatbot performance (*Table 2*). One RCT demonstrated that a chatbot with twice-daily text-based output for 2 weeks was associated with reduced opiate consumption compared with a control cohort (no messages received) following orthopaedic trauma surgery (26 opiate tablets *versus* 41 tablets)^15^. Another RCT found no differences in perceived quality of responses using the between chatbot *versus* real-time physician-written responses to user queries from women treated for breast cancer (average QLQ-INFO25 score 2.89 and 2.82 respectively)^16^. The third RCT reported no significant difference in symptoms of anxiety and depression, quantified using the Emotional Disturbance Anxiety Score, between patients using a chatbot (cohort 1) and a control cohort without chatbot access (cohort 2) over a 4-week study period. Upon completion of the first study period, the control cohort (cohort 2) were then granted access to the chatbot and symptoms of anxiety and depression were quantified after a second 4-week study period. After the second study period, patients in cohort 2 demonstrated a reduction in reported symptoms of anxiety compared with baseline measurements and anxiety scores after the first study period, although this reduction was not statistically significant^17^. A non-randomised comparative study demonstrated comparable follow-up consultation rates after orthopaedic surgery using a telephone-based conversational agent compared with calls made by individuals, saving an estimated 9.3 hours per 100 participants^18^.

**Table 2:** Technical details, acceptability criteria and outcomes assessed

Reference	Chatbot features	Device	Adherence	Other outcomes measured
**Anthony *et al.*, 2020^15^**	System-focused dialogue initiative Text output	Smartphone (text)	Not reported	**Postoperative pain** 36.5% reduction in number of opiate tablets used in intervention group (*P* = 0.004) 35% decrease in morphine milliequivalents consumed *versus* control (*P* = 0.006)
				**Patient-reported outcomes** Lower mean postoperative pain intensity 3 A PROMIS score in intervention arm (45.9 ± 7.2 *versus* 49.7 ± 8.8, *P* = 0.04) Lower mean postoperative pain interference 8 A PROMIS score in intervention arm (60.6 ± 8.2 *versus* 56.6 ± 9.4, *P* = 0.04)
**Bian *et al.*, 2020^18^**	Frame based Mixed dialogue initiative Spoken input/output	Smartphone (call)	92.2% follow-up rate (*versus* 93.3% in control)	**Patient feedback** 10.3% of patients contacted via chatbot provided feedback (*versus* 2.5% control)
				**Time per 100 patients** 0 *versus* 9.3 hours for chatbot and control respectively
**Bibault *et al.*, 2019^16^**	Frame based User-focused dialogue Text input and output	Web based or smartphone application	Not reported	**Quality of response** Perceived quality of response to the answers provided to user queries assessed using the QLQ-INFO25 (a patient-satisfaction score). Patients assessing chatbot responses gave a higher average rating compared with rating for responses given by physicians in real time. Success was defined as a score greater than or equal to 3 on a satisfaction scale of 1–4. Overall, non-inferiority was demonstrated between perceived quality of responses, however when individual items of the QLQ-INFO25 were assessed individually, non-inferiority of response satisfaction could not be demonstrated in 9 of 25 items
				**Patient satisfaction** 59% of patients wanted more information (*versus* 65% control) 85% of patients found information useful (*versus* 83.1% control) 85% of patients satisfied with amount of information received (*versus* 77% control)
**Black *et al.*, 2020^21^**	Mixed dialogue initiative Orientated to book follow-up appointments	Smartphone (application)	83.3% of participants engaged with the chatbot	**Patient experience** 60% highly satisfied (rated chatbot useful or very useful)
**Chaix *et al.*, 2019^24^**	Frame based User-focused dialogue Text input and output	Web based or smartphone application	31% retention rate after 8 months (N.B. only calculated for 956 patients)	**User response characteristics** Average response length 21.5 words
				**Patient experience** 93.95% overall patient satisfaction 88% stated that chatbot provided them with support and helped them follow their treatment effectively
**Giorgino *et al.*, 2005^20^**	Frame based Mixed dialogue initiative Spoken input/output Orientated to integrate with medical records	Smartphone (call)	Not reported	**Technical details** 80% consultation conclusion rate reached by system 18 questions per interaction Average consultation call time 3.3 minutes
**Goldenthal *et al.*, 2019^23^**	Frame based System-focused dialogue initiative Text input/output	Smartphone (application)	35% of participants engaged with chatbot	**Reasons for not activating chatbot** Misplacing instructions for chatbot use (*n* = 6), relying on follow-up with clinic or discharge materials (*n* = 4), inability to activate chatbot (*n* = 2) and inability to text (*n* = 1)
**Greer *et al.*, 2019^17^**	Frame based Mixed dialogue initiative Text input and output	Smartphone (Facebook messenger)	Mean of 12.1 sessions (73.8 minutes total engagement time) across 4 weeks *versus* 18.1 sessions (27.1 minutes total engagement time)	**Patient satisfaction** Patients rated chatbot as useful (average score 2/3) Patients likely to recommend chatbot to friend (average rating 6.9/10) **Anxiety and depression symptoms** Participants in intervention arm reported greater reduction in anxiety *versus* the control arm as per the PROMIS Emotional Distress-Anxiety Short Form (2.58 t-score units *versus* 0.7, *P* = 0.09) Both intervention and control arms reported a reduction in depressive symptoms as per the PROMIS Emotional Distress-Depression Short Form (1.83 *versus* 1.38, *P* = 0.77)
**Piau *et al.*, 2019^22^**	Rule based System-focused dialogue initiative Voice and text input/output	Smartphone (application)	86% compliance over study period	**Patient experience** 3 participants provided feedback (33%) All three were satisfied or very satisfied **Technical details** 3.5-minute average time to complete consultation All patients had a smartphone prior to recruitment
**Rhee *et al.*, 2014^19^**	Frame based Mixed dialogue initiative Text input/output	Smartphone (application)	81–97% response rate for system-initiated questions	**Patient experience** High overall satisfaction reported **Technical details** Average number of user imitated questions: 19

PROMIS, Patient Reported Outcomes Measurement Information System.

### Registered trials

The authors’ search found two additional registered protocols for ongoing clinical trials. Study protocols outline the intended use of chatbots to facilitate questionnaire completion at 6 and 8 months following bariatric surgery^26^ and for daily consultation with patients treated for Parkinson’s disease^27^ (*Fig.S3*, supplementary material).

## Discussion

The use of chatbots following a physical healthcare intervention is a new and evolving field, with eight of 10 studies published during or after 2019. It seems likely that this will continue to increase, with a move towards efficiency in healthcare systems and a move away from face-to-face follow-up arising from the COVID-19 pandemic.

A review investigating the broader use of conversational agents in healthcare has been published^4^, while the present review was focused on the role of technology after interventions. The systematic review identified 10 studies of different designs, mostly of moderate to poor quality. All outcome measures were inconsistently defined and outcome assessors were not blinded, predisposing to detection bias and Hawthorne effect. One study attempted to reduce this by blinding participants to responses from either the chatbot or physicians^16^, although by the nature of the intervention, a Hawthorne effect cannot be ignored.

Acceptability and patient experience using automated conversational agents was largely positive^19^^,^^21^. There was no clinically important difference in rates of patient satisfaction with chatbot responses compared with real-time physician-generated responses to user queries, measured using the QLQ-INFO25^16^. Previous work has demonstrated the QLQ-INFO25 is acceptable with good internal consistency and test–retest reliability^28^. The reduction in opiate prescribing, time and cost saving reported in one small study provides useful evidence supporting investment in automated follow-up systems^15^.

Despite the metrics used being heterogeneous, data around success of implementation suggest considerable variation. Some learning points were simple and applicable. One study described a 35 per cent interaction rate with their chatbot, with the primary reason for poor interaction being ‘misplacing instructions for chatbot use’^23^, while another demonstrated an initial engagement rate of 100 per cent at the start of the study that gradually fell to 31 per cent over 8 months^24^, likely to represent reduced enthusiasm for patient engagement, although it might represent patient adaptation to their current health state. Some support for the latter is that most (88 per cent) participants reported that the chatbot provided them with support and helped them follow their treatment plan. A structured sequence to implementation may increase success, and frameworks for this have been developed for the deployment of PROMs that might be applicable to automated follow-up systems^29^.

No study identified in the current systematic review examined patient safety. If autonomous agents are to be used in clinical practice to monitor patient status actively after intervention, rigorous safety testing using simulated patients is warranted before clinical adoption. Following implementation, prospective registries of technological adverse events should be kept. Here, technological adverse events refer to patient harm directly caused by technology. This harm may be direct (inappropriate clinical advice) or indirect (failing to identify clinical signs of deterioration). All studies identified in this systematic review deployed agents on mobile devices. In the UK, 70 per cent of adults own a smartphone and over half regularly use applications^30^. Disparities in socioeconomic status and technological literacy may limit access to healthcare. Future epidemiological studies should seek to ascertain whether clinical implementation of technologies negatively impacts the health of certain cohorts within the population.

The present study has a number of limitations. A small number of heterogeneous studies were identified, reporting a variety of different adherence and clinical-outcome measures. The majority of studies were small, non-comparative feasibility studies. The comparative studies were at risk of selection and detection bias owing to the nature of interventions and relative infancy of the field. Varying technical descriptions of agents were provided and heterogeneity in outcome reporting precluded meaningful meta-analysis, limiting the strength of conclusions that can safely be drawn.

There is, nevertheless, early evidence of uptake of automated conversational agents in the outpatient management of patients following physical healthcare interventions. Despite a range of chatbot builds and clinical uses, they seem to be generally acceptable, although effectiveness remains to be proven. Attention to practical details around deployment may improve implementation success of future systems.

## Acknowledgements

L.G. was involved in idea inception, search strategy design, data extraction, analysis and writing. A.S. and J.C.R.W. were involved in abstract screening and manuscript review. C.J.H., D.C. and M.G. critically reviewed the manuscript. J.B. and J.N.R. were involved in idea inception, search strategy design and manuscript review.

## Funding

No specific funding was received for the conduct of this review. J.B. is supported by National Institute for Health Research Capability Funding via University Hospitals Coventry and Warwickshire. C.J.H. is funded by a National Institute for Health Research (NIHR) Doctoral Research Fellowship (NIHR300684). J.N.R. is funded by an NIHR Postdoctoral Fellowship (PDF-2017-10-075). The views expressed are those of the authors and not necessarily those of the NHS, the NIHR or the Department of Health and Social Care.

*Disclosure*. The authors declare no conflicts of interest.

## Supplementary material

Supplementary material is available at *BJS Open* online

## Supplementary Material

zrab070_Supplementary_Data
