# Supplementary material for: Automated conversational agents for post-intervention follow-up: a systematic review
Source: BJS Open. 2021 Jul 29;5(4):zrab070. doi: 10.1093/bjsopen/zrab070 (PMC8320342; doi:10.1093/bjsopen/zrab070)
Supplement: zrab070_Supplementary_Data [file zrab070_supplementary_data.zip › Supplementary information.docx]

Supplementary Figure 1. Search terms

EMBASE

| \|  \| [# ▲](http://ovidsp.dc2.ovid.com.iclibezp1.cc.ic.ac.uk/ovid-b/ovidweb.cgi?&S=OHGIFPLPAHEBCLFJIPAKIFPECKCEAA00&Sort+Sets=descending) \| **Searches** \| **Results** \| \| --- \| --- \| --- \| --- \| \|  \| 1 \| chatbot*.mp. \| 159 \| \|  \| 2 \| (conversation* adj3 agent*).mp. \| 162 \| \|  \| 3 \| exp Artificial Intelligence/ \| 40961 \| \|  \| 4 \| exp Expert Systems/ \| 5420 \| \|  \| 5 \| sentiment analy*.mp. \| 455 \| \|  \| 6 \| exp Natural Language Processing/ \| 5439 \| \|  \| 7 \| Natural Language Process*.mp. \| 6679 \| \|  \| 8 \| post intervention*.mp. \| 26765 \| \|  \| 9 \| post operative*.mp. \| 147585 \| \|  \| 10 \| exp Specialties, Surgical/ \| 5329761 \| \|  \| 11 \| (health* adj3 intervention*).mp. \| 46373 \| \|  \| 12 \| exp Aftercare/ \| 1624952 \| \|  \| 13 \| exp Rehabilitation/ \| 418986 \| \|  \| 14 \| exp Telerehabilitation/ \| 872 \| \|  \| 15 \| Follow-up.mp. \| 2043234 \| \|  \| 16 \| exp Treatment Outcome/ \| 1682043 \| \|  \| 17 \| 8 or 9 or 10 or 11 \| 5417032 \| \|  \| 18 \| 12 or 13 or 14 or 15 or 16 \| 3628348 \| \|  \| 19 \| 1 or 2 or 3 or 4 or 5 or 6 or 7 \| 52069 \| \|  \| 20 \| 17 and 18 and 19 \| 471 \| |
| --- | --- | --- | --- | --- | --- | --- | --- | --- | --- | --- | --- | --- | --- | --- | --- | --- | --- | --- | --- | --- | --- | --- | --- | --- | --- | --- | --- | --- | --- | --- | --- | --- | --- | --- | --- | --- | --- | --- | --- | --- | --- | --- | --- | --- | --- | --- | --- | --- | --- | --- | --- | --- | --- | --- | --- | --- | --- | --- | --- | --- | --- | --- | --- | --- | --- | --- | --- | --- | --- | --- | --- | --- | --- | --- | --- | --- | --- | --- | --- | --- | --- | --- | --- | --- |

MEDLINE

|  | [# ▲](http://ovidsp.dc2.ovid.com.iclibezp1.cc.ic.ac.uk/ovid-b/ovidweb.cgi?&S=OHGIFPLPAHEBCLFJIPAKIFPECKCEAA00&Sort+Sets=descending) | **Searches** | **Results** |
| --- | --- | --- | --- |
|  | 1 | chatbot*.mp. | 139 |
|  | 2 | (conversation* adj3 agent*).mp. | 156 |
|  | 3 | exp Artificial Intelligence/ | 98956 |
|  | 4 | exp Expert Systems/ | 3405 |
|  | 5 | sentiment analy*.mp. | 364 |
|  | 6 | exp Natural Language Processing/ | 4321 |
|  | 7 | Natural Language Process*.mp. | 6225 |
|  | 8 | post intervention*.mp. | 15752 |
|  | 9 | post operative*.mp. | 70123 |
|  | 10 | exp Specialties, Surgical/ | 199478 |
|  | 11 | (health* adj3 intervention*).mp. | 37567 |
|  | 12 | exp Aftercare/ | 193126 |
|  | 13 | exp Rehabilitation/ | 305928 |
|  | 14 | exp Telerehabilitation/ | 389 |
|  | 15 | Follow-up.mp. | 1338663 |
|  | 16 | exp Treatment Outcome/ | 1059566 |
|  | 17 | 8 or 9 or 10 or 11 | 320855 |
|  | 18 | 12 or 13 or 14 or 15 or 16 | 2331150 |
|  | 19 | 1 or 2 or 3 or 4 or 5 or 6 or 7 | 101093 |
|  | 20 | 17 and 18 and 19 | 226 |

PSYCHINFO

| [# ▲](http://ovidsp.dc2.ovid.com.iclibezp1.cc.ic.ac.uk/ovid-b/ovidweb.cgi?&S=OHGIFPLPAHEBCLFJIPAKIFPECKCEAA00&Sort+Sets=descending) | **Searches** | **Results** | **Type** |  |
| --- | --- | --- | --- | --- |
|  | 1 | chatbot*.mp. | 80 |  |
|  | 2 | (conversation* adj3 agent*).mp. | 276 |  |
|  | 3 | exp Artificial Intelligence/ | 21447 |  |
|  | 4 | exp Expert Systems/ | 8762 |  |
|  | 5 | sentiment analy*.mp. | 522 |  |
|  | 6 | exp Natural Language Processing/ | 490 |  |
|  | 7 | Natural Language Process*.mp. | 1244 |  |
|  | 8 | post intervention*.mp. | 7523 |  |
|  | 9 | post operative*.mp. | 1965 |  |
|  | 10 | (health* adj3 intervention*).mp. | 17169 |  |
|  | 11 | exp Surgery/ | 71807 |  |
|  | 12 | FOLLOW-UP.mp. | 120518 |  |
|  | 13 | exp Aftercare/ | 1090 |  |
|  | 14 | exp Rehabilitation/ | 48272 |  |
|  | 15 | exp Telerehabilitation/ | 139 |  |
|  | 16 | 1 or 2 or 3 or 4 or 5 or 6 or 7 | 29232 |  |
|  | 17 | 8 or 9 or 10 or 11 | 96729 |  |
|  | 18 | 12 or 13 or 14 or 15 | 166356 |  |
|  | 19 | 16 and 17 and 18 | 10 |  |

CINAHL

| S20 | S17 AND S18 AND S19 | **Expanders** - Apply equivalent subjects  **Search modes** - Boolean/Phrase | [**View Results**](javascript:__doPostBack('ctl00$ctl00$FindField$FindField$historyControl$HistoryRepeater$ctl00$linkResults','')) (91)  [**View Details**](javascript:showShDetails(%22ctl00_ctl00_FindField_FindField_historyControl_ctrlPopup%22,%20%22S20%22);)  [**Edit**](http://web.a.ebscohost.com/Legacy/Views/UserControls/Ehost/) |  |
| --- | --- | --- | --- | --- |
|  | S19 | S12 OR S13 OR S14 OR S15 OR S16 | **Expanders** - Apply equivalent subjects  **Search modes** - Boolean/Phrase | [**View Results**](javascript:__doPostBack('ctl00$ctl00$FindField$FindField$historyControl$HistoryRepeater$ctl01$linkResults','')) (815,545)  [**View Details**](javascript:showShDetails(%22ctl00_ctl00_FindField_FindField_historyControl_ctrlPopup%22,%20%22S19%22);)  [**Edit**](http://web.a.ebscohost.com/Legacy/Views/UserControls/Ehost/) |
|  | S18 | S8 OR S9 OR S10 OR S11 | **Expanders** - Apply equivalent subjects  **Search modes** - Boolean/Phrase | [**View Results**](javascript:__doPostBack('ctl00$ctl00$FindField$FindField$historyControl$HistoryRepeater$ctl02$linkResults','')) (97,432)  [**View Details**](javascript:showShDetails(%22ctl00_ctl00_FindField_FindField_historyControl_ctrlPopup%22,%20%22S18%22);)  [**Edit**](http://web.a.ebscohost.com/Legacy/Views/UserControls/Ehost/) |
|  | S17 | S1 OR S2 OR S3 OR S4 OR S5 OR S6 OR S7 | **Expanders** - Apply equivalent subjects  **Search modes** - Boolean/Phrase | [**View Results**](javascript:__doPostBack('ctl00$ctl00$FindField$FindField$historyControl$HistoryRepeater$ctl03$linkResults','')) (18,482)  [**View Details**](javascript:showShDetails(%22ctl00_ctl00_FindField_FindField_historyControl_ctrlPopup%22,%20%22S17%22);)  [**Edit**](http://web.a.ebscohost.com/Legacy/Views/UserControls/Ehost/) |
|  | S16 | (MH "Treatment Outcomes+") | **Expanders** - Apply equivalent subjects  **Search modes** - Boolean/Phrase | [**View Results**](javascript:__doPostBack('ctl00$ctl00$FindField$FindField$historyControl$HistoryRepeater$ctl04$linkResults','')) (367,507)  [**View Details**](javascript:showShDetails(%22ctl00_ctl00_FindField_FindField_historyControl_ctrlPopup%22,%20%22S16%22);)  [**Edit**](http://web.a.ebscohost.com/Legacy/Views/UserControls/Ehost/) |
|  | S15 | follow up | **Expanders** - Apply equivalent subjects  **Search modes** - Boolean/Phrase | [**View Results**](javascript:__doPostBack('ctl00$ctl00$FindField$FindField$historyControl$HistoryRepeater$ctl05$linkResults','')) (248,665)  [**View Details**](javascript:showShDetails(%22ctl00_ctl00_FindField_FindField_historyControl_ctrlPopup%22,%20%22S15%22);)  [**Edit**](http://web.a.ebscohost.com/Legacy/Views/UserControls/Ehost/) |
|  | S14 | (MH "Telerehabilitation") | **Expanders** - Apply equivalent subjects  **Search modes** - Boolean/Phrase | [**View Results**](javascript:__doPostBack('ctl00$ctl00$FindField$FindField$historyControl$HistoryRepeater$ctl06$linkResults','')) (189)  [**View Details**](javascript:showShDetails(%22ctl00_ctl00_FindField_FindField_historyControl_ctrlPopup%22,%20%22S14%22);)  [**Edit**](http://web.a.ebscohost.com/Legacy/Views/UserControls/Ehost/) |
|  | S13 | (MH "Rehabilitation+") | **Expanders** - Apply equivalent subjects  **Search modes** - Boolean/Phrase | [**View Results**](javascript:__doPostBack('ctl00$ctl00$FindField$FindField$historyControl$HistoryRepeater$ctl07$linkResults','')) (288,878)  [**View Details**](javascript:showShDetails(%22ctl00_ctl00_FindField_FindField_historyControl_ctrlPopup%22,%20%22S13%22);)  [**Edit**](http://web.a.ebscohost.com/Legacy/Views/UserControls/Ehost/) |
|  | S12 | (MH "After Care") | **Expanders** - Apply equivalent subjects  **Search modes** - Boolean/Phrase | [**View Results**](javascript:__doPostBack('ctl00$ctl00$FindField$FindField$historyControl$HistoryRepeater$ctl08$linkResults','')) (14,454)  [**View Details**](javascript:showShDetails(%22ctl00_ctl00_FindField_FindField_historyControl_ctrlPopup%22,%20%22S12%22);)  [**Edit**](http://web.a.ebscohost.com/Legacy/Views/UserControls/Ehost/) |
|  | S11 | health* N3 intervention* | **Expanders** - Apply equivalent subjects  **Search modes** - Boolean/Phrase | [**View Results**](javascript:__doPostBack('ctl00$ctl00$FindField$FindField$historyControl$HistoryRepeater$ctl09$linkResults','')) (27,893)  [**View Details**](javascript:showShDetails(%22ctl00_ctl00_FindField_FindField_historyControl_ctrlPopup%22,%20%22S11%22);)  [**Edit**](http://web.a.ebscohost.com/Legacy/Views/UserControls/Ehost/) |
|  | S10 | (MH "Specialties, Surgical+") | **Expanders** - Apply equivalent subjects  **Search modes** - Boolean/Phrase | [**View Results**](javascript:__doPostBack('ctl00$ctl00$FindField$FindField$historyControl$HistoryRepeater$ctl10$linkResults','')) (44,271)  [**View Details**](javascript:showShDetails(%22ctl00_ctl00_FindField_FindField_historyControl_ctrlPopup%22,%20%22S10%22);)  [**Edit**](http://web.a.ebscohost.com/Legacy/Views/UserControls/Ehost/) |
|  | S9 | post operative* | **Expanders** - Apply equivalent subjects  **Search modes** - Boolean/Phrase | [**View Results**](javascript:__doPostBack('ctl00$ctl00$FindField$FindField$historyControl$HistoryRepeater$ctl11$linkResults','')) (14,094)  [**View Details**](javascript:showShDetails(%22ctl00_ctl00_FindField_FindField_historyControl_ctrlPopup%22,%20%22S9%22);)  [**Edit**](http://web.a.ebscohost.com/Legacy/Views/UserControls/Ehost/) |
|  | S8 | post intervention* | **Expanders** - Apply equivalent subjects  **Search modes** - Boolean/Phrase | [**View Results**](javascript:__doPostBack('ctl00$ctl00$FindField$FindField$historyControl$HistoryRepeater$ctl12$linkResults','')) (12,778)  [**View Details**](javascript:showShDetails(%22ctl00_ctl00_FindField_FindField_historyControl_ctrlPopup%22,%20%22S8%22);)  [**Edit**](http://web.a.ebscohost.com/Legacy/Views/UserControls/Ehost/) |
|  | S7 | Natural Language Process* | **Expanders** - Apply equivalent subjects  **Search modes** - Boolean/Phrase | [**View Results**](javascript:__doPostBack('ctl00$ctl00$FindField$FindField$historyControl$HistoryRepeater$ctl13$linkResults','')) (2,267)  [**View Details**](javascript:showShDetails(%22ctl00_ctl00_FindField_FindField_historyControl_ctrlPopup%22,%20%22S7%22);)  [**Edit**](http://web.a.ebscohost.com/Legacy/Views/UserControls/Ehost/) |
|  | S6 | (MH "Natural Language Processing") | **Expanders** - Apply equivalent subjects  **Search modes** - Boolean/Phrase | [**View Results**](javascript:__doPostBack('ctl00$ctl00$FindField$FindField$historyControl$HistoryRepeater$ctl14$linkResults','')) (1,795)  [**View Details**](javascript:showShDetails(%22ctl00_ctl00_FindField_FindField_historyControl_ctrlPopup%22,%20%22S6%22);)  [**Edit**](http://web.a.ebscohost.com/Legacy/Views/UserControls/Ehost/) |
|  | S5 | sentiment analy* | **Expanders** - Apply equivalent subjects  **Search modes** - Boolean/Phrase | [**View Results**](javascript:__doPostBack('ctl00$ctl00$FindField$FindField$historyControl$HistoryRepeater$ctl15$linkResults','')) (206)  [**View Details**](javascript:showShDetails(%22ctl00_ctl00_FindField_FindField_historyControl_ctrlPopup%22,%20%22S5%22);)  [**Edit**](http://web.a.ebscohost.com/Legacy/Views/UserControls/Ehost/) |
|  | S4 | (MH "Expert Systems") | **Expanders** - Apply equivalent subjects  **Search modes** - Boolean/Phrase | [**View Results**](javascript:__doPostBack('ctl00$ctl00$FindField$FindField$historyControl$HistoryRepeater$ctl16$linkResults','')) (506)  [**View Details**](javascript:showShDetails(%22ctl00_ctl00_FindField_FindField_historyControl_ctrlPopup%22,%20%22S4%22);)  [**Edit**](http://web.a.ebscohost.com/Legacy/Views/UserControls/Ehost/) |
|  | S3 | (MH "Artificial Intelligence+") | **Expanders** - Apply equivalent subjects  **Search modes** - Boolean/Phrase | [**View Results**](javascript:__doPostBack('ctl00$ctl00$FindField$FindField$historyControl$HistoryRepeater$ctl17$linkResults','')) (17,752)  [**View Details**](javascript:showShDetails(%22ctl00_ctl00_FindField_FindField_historyControl_ctrlPopup%22,%20%22S3%22);)  [**Edit**](http://web.a.ebscohost.com/Legacy/Views/UserControls/Ehost/) |
|  | S2 | conversation* N3 agent* | **Expanders** - Apply equivalent subjects  **Search modes** - Boolean/Phrase | [**View Results**](javascript:__doPostBack('ctl00$ctl00$FindField$FindField$historyControl$HistoryRepeater$ctl18$linkResults','')) (90)  [**View Details**](javascript:showShDetails(%22ctl00_ctl00_FindField_FindField_historyControl_ctrlPopup%22,%20%22S2%22);)  [**Edit**](http://web.a.ebscohost.com/Legacy/Views/UserControls/Ehost/) |
|  | S1 | chatbot* | **Expanders** - Apply equivalent subjects  **Search modes** - Boolean/Phrase | [**View Results**](javascript:__doPostBack('ctl00$ctl00$FindField$FindField$historyControl$HistoryRepeater$ctl19$linkResults','')) (120)  [**View Details**](javascript:showShDetails(%22ctl00_ctl00_FindField_FindField_historyControl_ctrlPopup%22,%20%22S1%22);)  [**Edit**](http://web.a.ebscohost.com/Legacy/Views/UserControls/Ehost/) |

**Supplementary Figure 2A**: Quality of included cohort studies assessed using the National Heart, Lung and Blood Institute Quality Assessment Tool for Observational Cohort and Cross-Sectional Studies.

| Reference | **Q1** | **Q2** | **Q3** | **Q4** | **Q5** | **Q6** | **Q7** | **Q8** | **Q9** | **Q10** | **Q11** | **Q12** | **Q13** | **Q14** | **Overall assessment** |
| --- | --- | --- | --- | --- | --- | --- | --- | --- | --- | --- | --- | --- | --- | --- | --- |
| Black et al., | - | - | - | - | - | - | - | - | - | - | - | - | - | - | - |
| Chaix et al., | Y | Y | NA | N | N | Y | Y | NA | Y | NA | N | NR | NR | N | Fair |
| Giorgino et al., | N | N | NA | NA | N | Y | Y | NA | N | NA | N | NR | NR | NA | Poor |
| Goldenthal et al., | N | Y | NA | Y | N | Y | Y | NA | Y | NA | N | N | NR | N | Fair |
| Piau et al., | Y | Y | NA | Y | N | Y | Y | NA | Y | NA | Y | N | NR | N | Fair |
| Rhee et al., | Y | Y | NA | Y | N | Y | Y | NA | Y | NA | Y | N | NR | N | Fair |
| *NA- Not applicable; NR- Not reported* | | | | | | | | | | | | | | | |

**Supplementary Figure 2B**: Risk of bias of included randomised controlled trials assessed using the revised Cochrane Risk of Bias Tool

|  | **Risk of bias domain (assessments for the effect of assignment to intervention)** | | | | | **Overall risk of bias** |
| --- | --- | --- | --- | --- | --- | --- |
| **Study** | 1. Randomization process | 1. Deviations from the intended interventions | 1. Missing outcome data | 1. Measurement of the outcome | 1. Selection of the reported result |  |
| Anthony et al., | Low | Some concerns | Low | High | Some concerns | Some concerns |
| Bibault et al., | Low | Low | Low | Some concerns | Low | Some concerns |
| Greer et al., | Some concerns | Some concerns | Low | High | Low | High |

**Supplementary Figure 2C**: Risk of bias of included non-randomised controlled trials assessed using the Cochrane Risk of Bias in non-randomised studies of interventions (ROBINS-I) tool

| **Study** | Bias due to confounding | Bias in selection of participants into the study | Bias in classification of interventions | Bias due to deviation from the intended intervention | Bias due to missing data | Bias in the selection of the reported result | **Overall risk of bias** |
| --- | --- | --- | --- | --- | --- | --- | --- |
| Bian et al., | Moderate | Serious | Low | Low | Low | Moderate | Moderate |

**Supplementary Figure 3**: Details of relevant registered clinical trials

| **CENTRAL identification number** | **Specialty** | **Health intervention** | **Study intervention** | **Comparator** | **Outcome measures** |
| --- | --- | --- | --- | --- | --- |
| CN-02181004 | General surgery | Bariatric surgery | Chatbot used to complete questionnaire form as an adjunct to tele-consultation at six and nine months after surgery | Face-to-face consultation at six and nine months after surgery | Composite follow-up quality score  Patient satisfaction |
| CN-02172801 | Neurology | Medical management of Parkinson’s disease | Chatbot used for daily consultations with patients for a total of five months | In person consultation once or twice a week with a physician | Sound parameters and facial expression during the interaction |
